# Physical therapists’ perspectives on using contextual factors in clinical practice: Findings from an Italian national survey

**DOI:** 10.1371/journal.pone.0208159

**Published:** 2018-11-30

**Authors:** Giacomo Rossettini, Alvisa Palese, Tommaso Geri, Mirta Fiorio, Luana Colloca, Marco Testa

**Affiliations:** 1 Department of Neuroscience, Rehabilitation, Ophthalmology, Genetics, Maternal and Child Health, University of Genova - Campus of Savona, Savona, Italy; 2 Department of Medical and Biological Sciences, University of Udine, Udine, Italy; 3 Department of Neurosciences, Biomedicine and Movement Sciences, University of Verona, Verona, Italy; 4 Department of Pain Translational Symptom Science, School of Nursing, University of Maryland, Baltimore, Maryland, United States of America; 5 Departments of Anesthesiology and Psychiatry, School of Medicine, University of Maryland, Baltimore, Maryland, United States of America; 6 Center to Advance Chronic Pain Research, University of Maryland, Baltimore, Maryland, United States of America; New York University, UNITED STATES

## Abstract

**Background:**

Contextual factors (CFs) represent a potential therapeutic tool to boost physiotherapy outcomes, triggering placebo effects. Nevertheless, no evidence about the use of CFs among physical therapists is currently available.

**Objective:**

To investigate the use of CFs and the opinion of Italian physical therapists specialized in Orthopaedic Manual Therapy (OMTs) on their therapeutic benefits.

**Design:**

An exploratory cross-sectional online survey.

**Methods:**

A 17-item questionnaire and 2 clinical vignettes assessed the perspective of OMTs on the adoption of CFs in daily clinical practice. The target population was composed of 906 OMTs. An online survey was performed in 2016 using SurveyMonkey Software. Data were analyzed by descriptive and inferential statistics.

**Results:**

A total of 558 volunteers (61.6% of the target OMT population) participated in the study. Half of the participants (52.0%) claimed to use CFs frequently in their practice. More of 50% of OMTs valued the therapeutic significance of CFs for different health problems as determined by a combined psychological and physiological effect. OMTs considered the use of CFs ethically acceptable when they exert beneficial therapeutic effects and their effectiveness has emerged in previous clinical experiences (30.6%). They disagreed on the adoption of CFs when they are deceptive (14.1%). Moreover, OMTs did not communicate the adoption of CFs to patients (38.2%), and CFs were usually used in addition to other interventions to optimize clinical responses (19.9%). Psychological mechanisms, patient’s expectation and conditioning were believed to be the main components behind CFs (7.9%).

**Limitations:**

Considering that the data collected were self-reported and retrospective, recall and response biases may limit the internal and external validity of the findings.

**Conclusions:**

OMTs used CFs in their clinical practice and believed in their therapeutic effect. The knowledge of CFs, placebo and nocebo mechanisms and their clinical effects should be included in physical therapists’ university studies.

## Introduction

Contextual factors (CFs) have been proposed in the scientific literature as an emerging topic[1]. These are multidimensional aspects of the therapeutic encounter (provider, patient, patient-provider relationship, treatment and setting)[2] capable of producing biological and psychological responses that can trigger positive or negative clinical outcomes by placebo and nocebo effects[3]. Placebo effects have been associated with the optimal use of CFs, whereas nocebo has been associated with a negative context surrounding the clinical encounter[4]. Different psychological theories based upon expectations and learning processes have been put forward as the fundamentals mechanisms of CFs effects, whilst specific neurotransmitters such as endogenous cholecystokinin, opioid, endocannabinoid, vasopressin, and dopamine have been documented as orchestrating the neurobiology behind their clinical effect[5].

Although the use of CFs as triggers of placebo and nocebo effects has been studied for many years in medicine, they have been introduced in physical therapy only recently [6]. Clinically, CFs symbolize the psycho-social component of the physiotherapy treatment capable of modulating patients’ symptoms[7,8]. Available randomized controlled trials have reported the positive effect of CFs on musculoskeletal conditions such as low back pain[9–12], neck pain[13] and shoulder pain[14]. Patients’ expectations with regard to a treatment[10,13], the physical therapist’s verbal suggestions associated with treatment[9,14], and the enhanced therapeutic alliance between the patient and the physical therapist[11,12] have all been documented as improving outcomes in different domains such as pain, disability, expectation and satisfaction[15–17].

Despite the increased interest in the use of CFs also in some clinical trials[9–14], no data have been published to date on physical therapists’ perspectives harnessing CFs in routine clinical practice. On the contrary, available surveys have investigated the use of placebos in specific groups of healthcare providers in Europe, America and the Middle East [18] documenting an overall use of them ranging from 17.0% to 80.0% among physicians[19–36], and from 51.0% to 100.0% among nurses[22,37–39].

Clinical implementation and perspectives about CFs use have been suggested as a priority field of investigation[18] in different professional healthcare groups, such as physical therapists[6]. In fact, physical therapists establish a one-to-one relationship with the patient, following the clinical pathway alongside and, more directly, influencing their experience and degree of satisfaction[40]. Among these professionals, physical therapists specialized in Orthopaedic Manual Therapy (OMTs) represent a professional group to be investigated because their clinical practice is widely pervaded by CFs[41]. Therefore, within this area of medicine we decided to explore the clinical behaviours, definition, frequency of use, beliefs, ethical and communication implications, circumstances of application and mechanism of actions of CFs in a nationwide sample of Italian OMTs.

## Materials and methods

### Design

A quantitative exploratory web-based cross-sectional survey herein reported according to the Checklist for Reporting Results of Internet E-Surveys (CHERRIES) guidelines[42] and to STrengthening the Reporting of OBservational Studies in Epidemiology (STROBE)[43] was performed at the University of Genoa (Italy) between October and December 2016. Ethical approval was obtained from the Liguria Clinical Experimental Ethics Committee (P.R.236REG2016, approved on 19/07/2016).

### Participants and setting

A nationwide sample of Italian physical therapists specialized in OMTs was the target population identified from the complete email database of the Master in Rehabilitation of Musculoskeletal Disorders (MRDM) of Genoa University (n = 906). This advanced educational program captures almost the totality of the Italian physical therapists specialized as OMTs[44]; moreover, it represents the oldest academic post-graduate program in manual therapy in Italy[45], based upon the standards established by the International Federation of Orthopaedic Manipulative Physical Therapists[46].

Within the established population, we included those OMTs who: a) had a valid e-mail account, b) understood the Italian language; and c) were working as clinicians at the time of the survey. Considering previous surveys on placebos which showed that a likely response rate would range from 40.0% to 60.0%[19–21,25,26,36], we expected approximately 363 to 544 overall responses from the population of 906 OMTs. The application of these predicted values to the formula for estimating the sample size for a single population proportion with the population proportion set at 50.0%, which is the most conservative value to be applied, produced a two-sided 95.0% confidence level within three to four percentage points of the true value and a relative standard error ranging from 2.7 to 4.1[47].

### Questionnaire development and pre-testing

A survey tool made of questions and clinical vignettes was developed using distinct and iterative steps[48]. Items from the existing surveys on placebo were extracted from the literature. Moreover, two clinical vignettes were derived and adapted from a recent survey on placebo[20]. Clinical vignettes represent written case scenarios on fictitious patients: they are adopted for measuring the clinical behavior of health providers by asking participants to report what their behavior would be[49,50].

The initial list included 17 questions and 2 clinical vignettes that were critically evaluated for face and content validity[48] by a panel of 6 experts with extensive experience in placebo and survey design (a physician, a psychologist, a nurse, and three physical therapists). These experts worked independently and then agreed on the final list by proving feedback on content accuracy, wording, question order and survey structure. Adjustments were progressively included by considering the feedback that emerged. When full agreement among experts was achieved, a preliminary version of the survey made of 17 questions and 2 clinical vignettes was self-administered and piloted in a convenient sample of 10 OMTs (North, n = 4; Centre, n = 3; South of Italy, n = 3).

Once the pilot stage was over, a telephone debriefing session was performed[48]. The panel of experts conducted one-to-one interviews among the sample of 10 OMTs on the possible difficulties encountered when doing the survey (e.g., identifying questions that required further explanation, wording that was too difficult to read or that respondents seemed to find confusing) and the OMTs’ experience in answering the questions. Overall, the outcome of the pilot stage was satisfactory; therefore, no changes nor comments were necessary. Namely, the sample reported that questions and clinical vignettes were not ambiguous; wording was easy and simple to be understood and the self-administered experience was good.

### Questionnaire implementation

A self-administered questionnaire (translated into English, S1 File and in original language S2 File) divided into 3 sections (A, B and C) was used. The socio-demographic variables were investigated by 2 open-ended questions (e.g., age) and 5 closed multiple-choice questions (e.g., gender, geographic region) in section A. Two clinical vignettes, structured as closed multiple-choice questions, were included in section B:

1. the first vignette was on the use of transcutaneous electrical nerve stimulation (TENS) in a patient with low back pain and high positive expectations towards this treatment based on previous encouraging experience. OMTs were asked to undertake a decision in this situation in which the use of TENS did not present contraindications and in absence of any evidence of efficacy;
2. the second vignette was focused on an in-patient clinical case with shoulder pain positively responding when the active TENS was replaced by a sham TENS. Additionally, OMTs were asked to draw a conclusion on the efficacy and effectiveness of sham TENS.

The last section (section C) lists 10 closed questions. More specifically, six questions were single-choice questions exploring the knowledge of CFs, including the definition (e.g., ‘How would you define the therapeutic role of CFs?’), the frequency of CF use (answers from ‘never’ to ‘many times’) and the case-by-case frequency of CF use (Likert from 0 ‘never’ to 4 ‘daily’, and ‘I was not aware of it was a CF capable to influence therapeutic outcome’). The section explored also participants’ CFs belief (Likert from 0 ‘not at all’ to 4 ‘a lot of’) and the potential beneficial effects of CFs (e.g., ‘What are the potential effects of CFs in the following health problems?’). In the remaining 4 questions, multiple responses were allowed to describe the ethical implications perceived in using CFs (e.g., ‘The use of CFs for therapeutic purposes can be considered ethically acceptable when.…’), communication implications about CFs (e.g., ‘How do you communicate to the patient the use of CFs at the end of treatment?’), the circumstances under which they are applied (e.g., ‘Under what circumstances would you use CFs?’), and the possible mechanisms of action (e.g., ‘What mechanism of action can explain the effect of CFs?’). Overall, the term ‘contextual factor’ was preferred to ‘placebos’, as suggested in previous studies[26, 50].

### Data collection procedure

The SurveyMonkey (Survey-Monkey, Palo Alto, California, www.surveymonkey.com) online survey tool was used. The survey was administered over an eight-week period between 14^th^ October 2016 and 14^th^ December 2016. After permission was obtained from MRDM of Genova University, all OMTs were contacted by blast email [48]. An email containing the survey and a brief note outlining (a) the aim of the study, (b) data handling (anonymity), (c) the informed consent statement, and (d) the invitation to complete the survey, was delivered. Specifically, the statement within the email informed that by clicking on the survey link, respondents were providing their consent to participate in the study[48].

Two email reminders were sent 2 and 4 weeks after the initial contact to encourage those who did not participate in the survey. 10 to 15 minutes were needed to complete the survey, corresponding to the completion time found to optimize response rates in online surveys[51]. Participation was voluntary and no incentives were offered to participants; there was the option to decline to answer specific questions or to leave the entire questionnaire blank[48]. Participants were able to review or change responses using a back button before submitting their answers.

Data were downloaded and stored in an encrypted computer, and only the project manager had access to the information during all stages of the study. Participants were ensured that their identities would not be disclosed to investigators. All data were de-identified (name and email address) to maintain confidentiality and data protection[48].

### Data analysis

Survey data were downloaded from SurveyMonkey into Excel spreadsheets and reviewed for accuracy and missing values. A questionnaire was considered incomplete if > 20.0% of data were missing [52].

For questions allowing only one choice, descriptive statistics (mean, standard deviation) were used for continuous variables by calculating also confidence intervals (CI) at 95%, while absolute frequencies and percentages were applied to dichotomous, nominal, and ordinal variables. Age and years of clinical experience were transformed into ordinal variables considering a decade as variable levels for the analysis of correlations, as described below. For those questions with more than one choice, absolute frequency and percentages were calculated for every combination of responses given by each participant. For example, considering that the fields (n) asked in the domain ‘Ethic’ were four with dichotomous responses (r), we did not calculate the absolute frequency of the four possible fields but of their 16 combinations, given by the formula r^∧^n, to better describe the responses given by each participant.

The presence of any relationship between the individual characteristics (section A of the survey) and the responses given (sections B and C of the survey) was investigated with Cramer’s V which is a measure of strength and direction of association derived from chi-square statistics. Only correlation values higher > 0.60 were deemed acceptable and, therefore, here reported.

The five response options for the domains of frequency of use (‘never’, ‘around once per year’, ‘around once per month’, ‘around once per week’, ‘daily’) and beliefs about CFs (‘not at all’, ‘few’, ‘enough’, ‘much’, ‘a lot of’) were converted into a five-point Likert scale ranging from 0 (‘never’ and ‘not at all’) to 4 (‘daily’ and ‘very much’) in order to have an average distribution of the two domains and to analyze the relationship between the frequency of use and the associated beliefs about CFs using Spearman’s rho. R software[53] was used for data analysis with the packages psych[54] and ggplot2[55].

## Results

### Participant’s characteristics

Out of the 906 invited OMTs, a total of 571 responded (63.0%). Thirteen incomplete surveys were excluded from data analysis, leaving 558 questionnaires to be considered as valid (61.6%) for the analysis. The majority of OMTs (n = 329; 59.0%; 95%CI 54.7–63.0) were male, and their average age was 30.5 ± 6.5 years. 72% of participants (n = 400; 95%CI 67.7–75.3) lived in the North of Italy.

Participants reported a mean of 6.8 ± 5.7 years of clinical experience. A high proportion worked 31–45 hours/week (n = 316; 56.6%; 95%CI 52.4–60.8) as private practitioners (n = 433; 77.6%; 95%CI 73.9–80.9) in the musculoskeletal field (n = 472; 84.6%; 95%CI 81.3–87.4). The respondents’ demographics are described in Table 1.

**Table 1:** Participant characteristics (n = 558).

Demographic	Values	95%CI
*Gender*		
• Male, n (%)	329 (59.0)	54.7–63.0
• Female, n (%)	229 (41.0)	36.9–45.3
*Years*, average (SD)	30.5 (6.5)	30.0–31.1
*Italian Region*		
• North, n (%)	400 (71.7)	67.7–75.3
• Centre, n (%)	120 (21.5)	18.2–25.2
• South, n (%)	38 (6.8)	4.9–9.3
*Years of clinical practice*, average (SD)	6.8 (5.7)	6.3–7.3
*Workplace*, n (%)		
• Private practice	433 (77.6)	73.9–80.9
• Hospital	87 (15.6)	12.7–18.9
• Residential care (nursing home)	38 (6.8)	4.9–9.3
*Field of work*, n (%)		
• Musculoskeletal	472 (84.6)	81.3–87.4
• Geriatric	45 (8.1)	6.0–10.7
• Neurological	36 (6.4)	4.6–8.9
• Other: Hearth, Respiratory, Pediatric	5 (0.9)	0.3–2.2
*Hours of work per week*, n (%)		
• 0–15	26 (4.7)	3.1–6.8
• 16–30	102 (18.3)	15.2–21.8
• 31–45	316 (56.6)	52.4–60.8
• 46–60	102 (18.3)	15.2–21.8
• > 60	12 (2.1)	1.2–3.8

N, number of participants; %, percentage; SD, standard deviation; 95%CI, 95% confidence interval; >, more

### Definition of CFs

The majority of OMTs defined CFs as ‘an intervention without a specific effect for the condition being treated, but with a possible aspecific effect’ (n = 407; 72.9%; 95%CI 69.0–76.5). Instead, the minority of OMTs identified CFs as ‘a sham treatment used as control tests for safety and efficacy of active treatment’ (n = 20; 3.6%; 95%CI 2.3–5.6) and as ‘a harmless or inert intervention’ (n = 19; 3.4%; 95%CI 2.1–5.4).

The remaining participants considered CFs as ‘an intervention that has a special effect through known physiological mechanisms’ (n = 112; 20.1%; 95%CI 16.9–23.7).

### Clinical vignette 1

**Fig 1:**
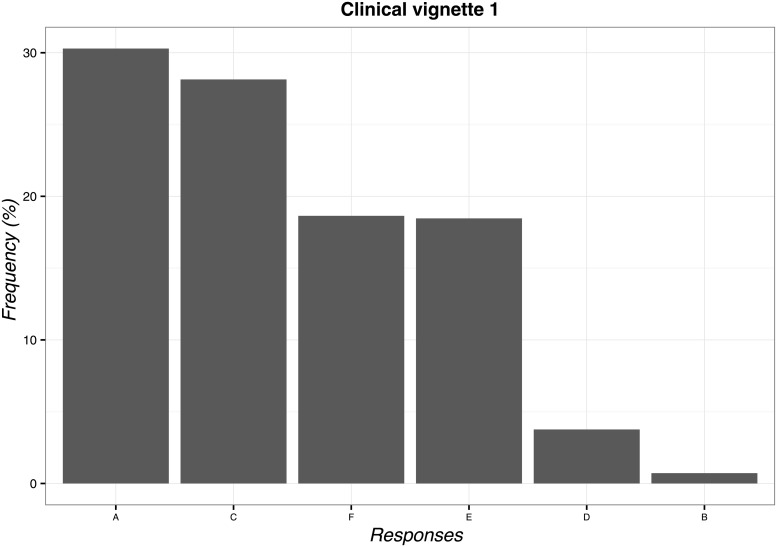
Percentages of responses for clinical vignette 1. A: to deliver TENS, B: to tell the patient that low back pain would resolve itself in a few days, C: to suggest the possibility of delivering TENS if the clinical condition fails to improve, D: to advise a follow-up appointment in the following days, E: to advise a different treatment commonly used for low back pain, F: try to convince the patient of the uselessness of TENS.

The most frequently chosen solution to the first vignette was ‘to deliver TENS’ (n = 169; 30.3%; 95%CI 26.5–34.3). The least frequent answer instead was ‘to tell the patient that low back pain would resolve itself in a few days’ (n = 4; 0.7%; 95%CI 0.2–1.9). The overall overview of data is reported in Fig 1.

### Clinical vignette 2

**Fig 2:**
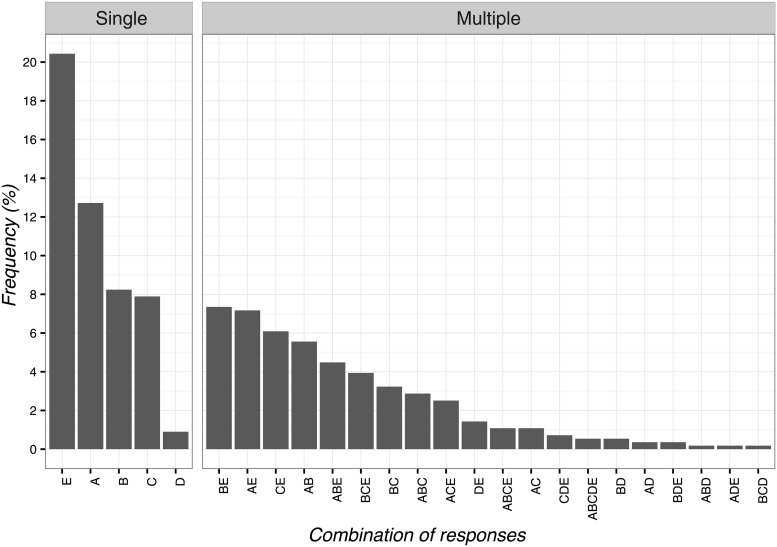
Percentages of responses for clinical vignette 2. A: pain is not organic but psychological, B: the patient is very suggestible, C: the natural decrease of pain intensity, D: the patient provides the response expected by the physical therapist, E: the positive attention of the healthcare team leads to decreased pain.

The most frequent answer to the second vignette was: ‘the positive attention of the healthcare team leads to decreased pain’ (n = 114; 20.4%; 95%CI 17.2–24.1), while the least frequent one was ‘the patient provides the response expected by the physical therapist’ (n = 5; 0.9%; 95%CI 0.3–2.2). Globally, the single items and their combinations are presented in Fig 2.

### Frequency of use

The frequency of use presented a mean of 3.04 (95%CI 3.00–3.07) on a five-point Likert scale, indicating a higher adoption of CFs among participant OMTs. Overall, 52% of OMTs (n = 290; 95%CI 47.7–56.2) claimed to use the CFs ‘many times’ in their clinical practice. The remaining reported the use as ‘often’ (n = 112; 20.1%; 95%CI 16.9–23.7), ‘at least once’ (n = 126; 22.6%; 95%CI 19.2–26.3), and ‘never’ (n = 30; 5.4%; 95%CI 3.7–7.7).

As for the specific adoption of CFs, the most used CFs were: ‘verbal communication’ (mean = 3.6; 95%CI 3.5–3.7), ‘patient-centered approach’ (mean = 3.6; 95%CI 3.5–3.7) and ‘empathetic therapeutic alliance with the patient’ (mean = 3.6; 95%CI 3.5–3.7). The least used CFs were (in descending order): an ‘adequate environmental architecture’ (mean = 2.4; 95%CI 2.2–2.5), ‘adequate design’ (mean = 2.2; 95%CI 2.1–2.4), the ‘uniform’ (mean = 2.2; 95%CI 2.0–2.3) and the ‘professional reputation’ (mean = 1.7; 95%CI 1.6–1.9). A complete report on CF use is presented in Table 2.

**Table 2:** Contextual factors use in clinical practice (n = 558).

Contextual factors items^a^	Likert Score mean (95%CI)	4 n (%); 95%CI	3 n (%); 95%CI	2 n (%); 95%CI	1 n (%); 95%CI	0 n (%); 95%CI	Unaware n (%); 95%CI
**A: Professional reputation** (e.g., qualification, expertise)	1.7 (1.6–1.9)	90 (16.1); 13.2–19.5	98 (17.6); 14.5–21.0	104 (18.6); 15.5–22.2	42 (7.5); 5.5–10.1	188 (33.7); 29.8–37.8	36 (6.4); 4.6–8.9
**A: Uniform** (e.g., white coat)	2.2 (2.0–2.3)	215 (38.5); 34.5–42.7	47 (8.4); 6.3–11.1	33 (5.9); 4.2–8.3	30 (5.4); 3.7-5-7	180 (32.3); 28.4–36.3	53 (9.5); 7.3–12.3
**A: Positive attitudes and optimistic behavior** (e.g., towards a patient’s dysfunctions)	3.5 (3.4–3.6)	416 (74.5); 70.7–78.1	80 (14.3); 11.6–17.6	19 (3.4); 2.1–5.4	9 (1.6); 0.8–3.1	32 (5.7); 4.0–8.1	2 (0.4); 0.1–1.4
**B: Patient’s expectation and preference** (e.g., towards a physiotherapy treatment)	3.1 (3.0–3.2)	277 (49.6); 45.4–53.9	173 (31.0); 27.2–35.0	55 (9.9); 7.6–12.7	15 (2.7); 1.6–4.5	37 (6.6); 4.8–9.1	1 (0.2); 0.0–1.2
**B: Patient’s previous experience** (e.g., towards a physiotherapy treatment)	2.8 (2.7–2.9)	183 (32.8); 28.9–36.9	197 (35.3); 31.4–39.4	93 (16.7); 13.7–20.1	31 (5.6); 3.9–7.9	48 (8.6); 6.5–11.3	6 (1.1); 0.4-2-4
**C: Verbal communication** (e.g., positive messages associated with the treatment)	3.6 (3.5–3.7)	465 (83.3); 79.9–86.3	44 (7.9); 5.8–10.5	15 (2.7); 1.6–4.5	1 (0.2); 0.0–1.2	32 (5.7); 4.0–8.1	1 (0.2); 0.0–1.2
**C: Non-verbal communication** (e.g., posture, gestures, eye contact, facial expressions)	3.4 (3.3–3.5)	374 (67.0); 62.9–70.9	107 (19.2); 16.0–22.7	20 (3.6); 2.3–5.6	11 (2.0); 1.0–3.6	42 (7.5); 5.5–10.1	4 (0.7); 0.2–2.0
**C: Empathetic therapeutic alliance with the patient** (e.g., active listening)	3.6 (3.5–3.7)	437 (78.3); 74.6–81.6	69 (12.4); 9.8–15.4	19 (3.4); 2.1–5.4	1 (0.2); 0.0–1.2	31 (5.6); 3.9–7.9	1 (0.2); 0.0–1.2
**D: Overt therapy** (e.g., possibility for the patient to see the therapy using a mirror)	3.0 (2.9–3.2)	288 (51.6); 47.4–55.8	128 (22.9); 19.6–26.7	50 (9.0); 6.8–11.7	17 (3.0); 1.8–4.9	59 (10.6); 8.2–13.5	16 (2.9); 1.7–4.7
**D: Patient-centered approach** (e.g., shared-decision of physiotherapy treatment)	3.6 (3.5–3.7)	451 (80.8); 77.3–84.0	59 (10.6); 8.2–13.5	14 (2.5); 1.4–4.3	2 (0.4); 0.1–1.4	32 (5.7); 4.0–8.1	0 (0.0); 0.0–0.0
**D: Professional approach to patient** (e.g., privacy, punctuality)	3.4 (3.4–3.5)	385 (69.0); 65.0–72.8	103 (18.5); 15.4–22.0	25 (4.5); 3.0–6.6	11 (2.0); 1.0–3.6	30 (5.4); 3.7–7.7	4 (0.7); 0.2–2.0
**D: Physical contact with the patient** (e.g., touch to inform, assist, prepare, take care)	3.5 (3.4–3.6)	414 (74.2); 70.3–77.7	82 (14.7); 11.9–18.0	16 (2.9); 1.7–4.7	7 (1.2); 0.5–2.7	31 (5.6); 3.9–7.9	8 (1.4); 0.7–2.9
**E: Comfortable setting** (e.g., little noise, music, fragrances, temperature)	3.1 (3.0–3.2)	327 (58.6); 54.4–62.7	99 (17.7); 14.7–21.2	44 (7.9); 5.8–10.5	15 (2.7); 1.6–4.5	64 (11.5); 9.0-14-5	9 (1.6); 0.8–3.2
**E: Adequate environmental architecture** (e.g., windows and skylights, supportive indicators)	2.4 (2.2–2.5)	219 (39.2); 35.2–43.4	75 (13.4); 10.8–16.6	56 (10.0); 7.7–12.9	22 (3.9); 2.5–6.0	147 (26.3); 22.8–30.2	39 (7.0); 5.1–9.5
**E: Adequate design** (e.g., decorations, ornaments and colors)	2.2 (2.1–2.4)	207 (37.1); 33.1–41.3	74 (13.3); 10.6–16.4	52 (9.3); 7.1–12.1	26 (4.7); 3.1–6.8	167 (29.9); 26.2–33.9	32 (5.7); 4.0-8-1

%, percentage; n, number of participants; 95%CI, 95% confidence interval; 0, never; 1, around once per year; 2, around once per month; 3, around once per week; 4, daily; A: physical therapist domain; B: patient domain; C: physical therapist—patient relationship domain; D: therapy domain; E: healthcare setting domain.

^**a**^ The items were reported from: Testa M, Rossettini G. Enhance placebo, avoid nocebo: How contextual factors affect physiotherapy outcomes. *Man Ther*. 2016;24:65–74.

Three CFs presented a bimodal response modality in terms of ‘daily’ (the ‘uniform’: n = 215; 38.5%; 95%CI 34.5–42.7; an ‘adequate environmental architecture’: n = 219; 39.2%; 95%CI 35.2–43.4; ‘an adequate design’: n = 207; 37.1%; 95%CI 33.1–41.3) and ‘never’ (‘the uniform’: n = 180; 32.3%; 95%CI 28.4–36.3; an ‘adequate environmental architecture’: n = 147; 26.3%; 95%CI 22.8–30.2; an ‘adequate design’: n = 167; 29.9%; 95%CI 26.2–33.9).

An analysis on the participants’ characteristics performed to understand the reason causing this kind of bimodal distribution revealed dependence on the workplace. In particular, the OMTs working in the private sector at the time of the survey used these CFs more frequently (an ‘adequate environmental architecture’: mean = 2.5, 95%CI 2.4–2.6; an ‘adequate design’: mean = 3.2, 95%CI 3.1–3.3) as compared to OMTs working in hospitals (an ‘adequate environmental architecture’: mean = 2.0, 95%CI 1.8–2.1; an ‘adequate design’: mean = 2.8, 95%CI 2.7–2.9) and in residential care settings (an ‘adequate environmental architecture’: mean = 2.0, 95%CI 1.8–2.1; an ‘adequate design’: mean = 2.7, 95%CI 2.5–2.8).

Moreover, a more frequent use of ‘the uniform’ was adopted by OMTs working in the private sector (mean = 2.2, 95%CI 2.1–2.4) and in hospitals (mean = 2.1, 95%CI 2.0–2.3) as compared to those who were working in residential care (mean = 1.7, 95%CI 1.6–1.8).

### Beliefs

The mean score of beliefs was 2.79 out of 5 (95%CI 2.77–2.82), thus denoting a substantial level of conviction towards CFs among OMTs.

In detail, the most believed CFs were (in descending order): ‘the empathetic therapeutic alliance with the patient’ (mean = 3.5; 95%CI 3.4–3.6), ‘the patient-centered approach’ (mean = 3.4; 95%CI 3.4–3.5), ‘the verbal communication’ (mean = 3.3; 95%CI 3.3–3.4). The least believed CFs were (in descending order): ‘the professional reputation’ (mean = 2.4; 95%CI 2.3–2.5), ‘the adequate environmental architecture’ (mean = 2.2; 95%CI 2.1–2.3), ‘the adequate design’ (mean = 2.1; 95%CI 2.0–2.2), and ‘the uniform’ (mean = 1.6; 95%CI 1.5–1.7). An overall description of beliefs towards CFs is presented in Table 3.

**Table 3:** Beliefs regarding contextual factors (n = 558).

Contextual factors items^a^	Likert Score mean (95%CI)	4 n (%); 95%CI	3 n (%); 95%CI	2 n (%); 95%CI	1 n (%); 95%CI	0 n (%); 95%CI	Unknow n (%); 95%CI
**A: Professional reputation** (e.g., qualification, expertise)	2.4 (2.3–2.5)	79 (14.2); 11.4–17.4	171 (30.6); 26.9–34.7	222 (39.8); 35.7–44.0	66 (11.8); 9.3–14.9	8 (1.4); 0.7–2.9	12 (2.1); 1.2–3.8
**A: Uniform** (e.g., white coat)	1.6 (1.5–1.7)	10 (1.8); 0.9–3.4	76 (13.6); 10.9–16.8	199 (35.7); 31.7–39.8	203 (36.4); 32.4–40.5	58 (10.4); 8.0–13.3	12 (2.1); 1.2–3.8
**A: Positive attitudes and optimistic behavior** (e.g., towards a patient’s dysfunctions)	3.1 (3.1–3.2)	203 (36.4); 32.4–40.5	238 (42.6); 38.5–46.9	97 (17.4); 14.4–20.8	18 (3.2); 2.0–5.1	0 (0); 0.0–0.8	2 (0.4); 0.1–1.4
**B: Patient’s expectation and preference** (e.g., towards a physiotherapy treatment)	3.1 (3.0–3.1)	189 (33.9); 30.0–38.0	240 (43.0); 38.9–47.2	107 (19.2); 16.0–22.7	18 (3.2); 2.0–5.1	2 (0.4); 0.1–1.4	2 (0.4); 0.1–1.4
**B: Patient’s previous experience** (e.g., towards a physiotherapy treatment)	2.8 (2.7–2.9)	122 (21.9); 18.5–25.6	244 (43.7); 39.6–48.0	147 (26.3); 22.8–30.2	36 (6.4); 4.6–8.9	3 (0.5); 0.1–1.7	6 (1.1); 0.4–2.4
**C: Verbal communication** (e.g., positive messages associated with the treatment)	3.3 (3.3–3.4)	266 (47.7); 43.5–51.9	219 (39.2); 35.2–43.4	54 (9.7); 7.4–12.5	11 (2.0); 1.0–3.6	1 (0.2); 0.0–1.2	7 (1.2); 0.5–2.7
**C: Not verbal communication** (e.g., posture, gestures, eye contact, facial expressions)	3.1 (3.1–3.2)	205 (36.7); 32.7–40.9	236 (42.3); 38.2–46.5	85 (15.2); 12.4–18.5	19 (3.4); 2.1–5.4	2 (0.4); 0.1–1.4	11 (2.0); 1.0–3.6
**C: Empathetic therapeutic alliance with the patient** (e.g., active listening)	3.5 (3.4–3.6)	332 (59.5); 55.3–63.6	175 (31.4); 27.6–35.4	36 (6.4); 4.6–8.9	10 (1.8); 0.9–3.4	0 (0); 0.0–0.8	5 (0.9); 0.3–2.2
**D: Overt therapy** (e.g., possibility for the patient to see the therapy using a mirror)	2.6 (2.5–2.7)	102 (18.3); 15.2–21.8	213 (38.2); 34.1–42.4	161 (28.8); 25.2–32.8	38 (6.8); 4.9–9.3	20 (3.6); 2.3–5.6	24 (4.3); 2.8–6.4
**D: Patient-centered approach** (e.g., shared-decision of physiotherapy treatment)	3.4 (3.4–3.5)	312 (55.9); 51.7–60.1	194 (34.8); 30.8–38.9	40 (7.2); 5.2-9-7	10 (1.8); 0.9–3.4	0 (0); 0.0–0.8	2 (0.4); 0.1–1.4
**D: Professional approach to patient** (e.g., privacy, punctuality)	2.7 (2.6–2.8)	108 (19.3); 16.2–22.9	239 (42.8); 38.7–47.1	157 (28.1); 24.5–32.1	41 (7.3); 5.4–9.9	6 (1.1); 0.4–2.4	7 (1.2); 0.5–2.7
**D: Physical contact with the patient** (e.g., touch to inform, assist, prepare, take care)	3.0 (3.0–3.1)	158 (28.3); 24.6–32.3	274 (49.1); 44.9–53.3	75 (13.4); 10.8–16.6	26 (4.7); 3.1–6.8	1 (0.2); 0.0–1.2	24 (4.3); 2.8–6.4
**E: Comfortable setting** (e.g., little noise, music, fragrances, temperature)	2.6 (2.5–2.6)	65 (11.6); 9.2–14.7	230 (41.2); 37.1–45.4	201 (36.0); 32.1–40.2	33 (5.9); 4.2–8.3	10 (1.8); 0.9–3.4	19 (3.4); 2.1–5.4
**E: Adequate environmental architecture** (e.g., windows and skylights, supportive indicators)	2.2 (2.1–2.3)	38 (6.8); 4.9–9.3	162 (29.0); 25.3–33.0	217 (38.9); 34.8–43.1	92 (16.5); 13.6–19.9	15 (2.7); 1.6–4.5	34 (6.1); 4.3–8.5
**E: Adequate design** (e.g., decorations, ornaments and colors)	2.1 (2.0–2.2)	35 (6.3); 4.5–8.7	134 (24.0); 20.6–27.8	236 (42.3); 38.2–46.5	101 (18.1); 15.0–21.6	18 (3.2); 2.0–5.1	34 (6.1); 4.3–8.5

%, percentage; n, number of participants; 95%CI, 95% confidence interval; 0, not at all; 1, few; 2, enough; 3, much; 4, a lot of; A: physical therapist domain; B: patient domain; C: physical therapist—patient relationship domain; D: therapy domain; E: healthcare setting domain.

^**a**^ The items were reported from: Testa M, Rossettini G. Enhance placebo, avoid nocebo: How contextual factors affect physiotherapy outcomes. *Man Ther*. 2016;24:65–74.

### Therapeutic effect

Overall, ‘physiological and psychological’ effects were the most chosen by OMTs caring after various health problems such as chronic pain (n = 436, 78.1%; 95%CI 74.4–81.4) and insomnia (n = 345; 61.8%; 95%CI 57.6–65.8). The ‘psychological’ effect was predominantly reported for oncological (n = 274; 49.1%; 95%CI 44.9–53.3) and emotional disorders (n = 232; 41.6%; 95%CI 37.5–45.8). OMTs identified as ‘no benefit’ the therapeutic effects behind several health conditions such as infectious (n = 229; 41.0%; 95%CI 36.9–45.3) and immune/allergic problems (n = 167; 29.9%; 95%CI 26.2–33.9). No health problem was selected for having an exclusively ‘physiological’ effect. An overall report of therapeutic effects is presented in Table 4.

**Table 4:** Therapeutic effect(s) of contextual factors (n = 558).

Clinical conditions	Psychological and Physiological n (%); 95%CI	Physiological n (%); 95%CI	Psychological n (%); 95%CI	No benefit n (%); 95%CI
Chronic pain	436 (78.1); 74.4–81.4	12 (2.1); 1.2–3.8	104 (18.6); 15.5–22.2	6 (1.1); 0.4–2.4
Insomnia	345 (61.8); 57.6–65.8	4 (0.7); 0.2–1.9	167 (29.9); 26.2–33.9	42 (7.5); 5.5–10.1
Acute pain	317 (56.8); 52.6–60.9	26 (4.7); 3.1–6.8	164 (29.4); 25.7–33.4	51 (9.1); 6.9–11.9
Cognitive disorder	317 (56.8); 52.6–60.9	8 (1.4); 0.7–2.9	230 (41.2); 37.1–45.4	3 (0.5); 0.1–1.7
Rheumatologic problem	313 (56.1); 51.9–60.2	19 (3.4); 2.1–5.4	170 (30.5); 26.7–34.5	56 (10.3); 7.7–12.9
Gastrointestinal problem	307 (55); 50.8–59.2	21 (3.8); 2.4–5.8	112 (20.1); 16.9–23.7	118 (21.1); 17.9–24.8
Emotional disorder	303 (54.3); 50.1–58.5	10 (1.8); 0.9–3.4	232 (41.6); 37.5–45.8	13 (2.3); 1.3–4.1
Sexual problem	295 (52.9); 48.6–57.1	9 (1.6); 0.8–3.1	151 (27.1); 23.5–31.0	103 (18.5); 15.4–22.0
Neurological problem	289 (51.8); 47.6–56.0	10 (1.8); 0.9–3.4	155 (27.8); 24.1–31.7	104 (18.6); 15.5–22.2
Cardiovascular problem	253 (45.3); 41.2–49.6	20 (3.6); 2.3–5.6	156 (28.0); 24.3–31.9	129 (23.1); 19.7–26.9
Drug and medication addiction	238 (42.6); 38.5–46.9	7 (1.2); 0.5–2.7	187 (33.5); 29.6–37.6	126 (22.6); 19.2–26.3
Immune/allergic problem	204 (36.6); 32.6–40.7	17 (3.0); 1.8–4.9	170 (30.5); 26.7–34.5	167 (29.9); 26.2–33.9
Oncological problem	195 (34.9); 31.0–39.1	7 (1.2); 0.5–2.7	274 (49.1); 44.9–53.3	82 (14.7); 11.9–17.8
Infectious problem	123 (22.0); 18.7–25.8	17 (3.0); 1.8–4.9	189 (33.9); 30.0–38.0	229 (41.0); 36.9–45.3

%, percentage; n, number of participant; 95%CI, 95% confidence interval.

### Ethical implications

**Fig 3:**
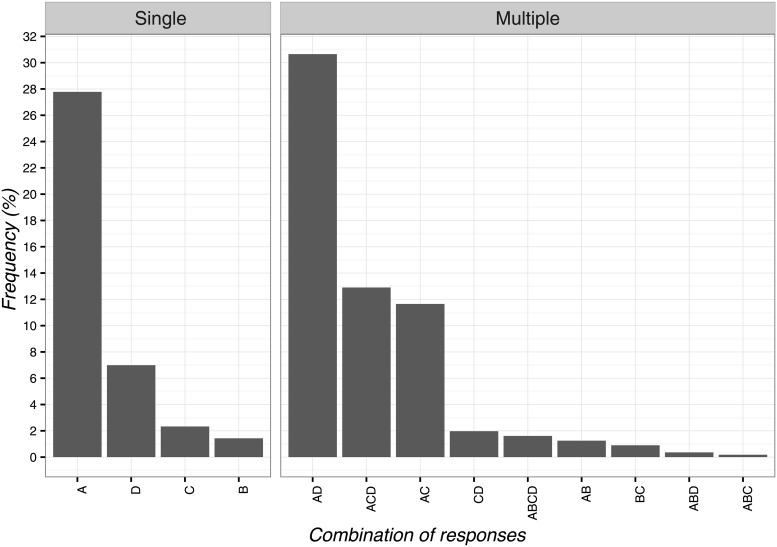
Percentages of responses for ethical use of contextual factors. A: it exerts beneficial psychological effects, B: the other therapies are over, C: the patient wants or expects this treatment, D: effectiveness shown by clinical experience.

The most frequent response on the ethical use of CFs was ‘it exerts beneficial psychological effects’ (n = 155; 27.8%; 95%CI 24.1–31.7) and its combination with the response ‘clinical experience has shown the effectiveness’ (Fig 2). The least selected item was ‘the other therapies are over’ (n = 8; 1.4%; 95%CI 0.7–2.9) and its combinations with the other items as presented in Fig 3.

**Fig 4:**
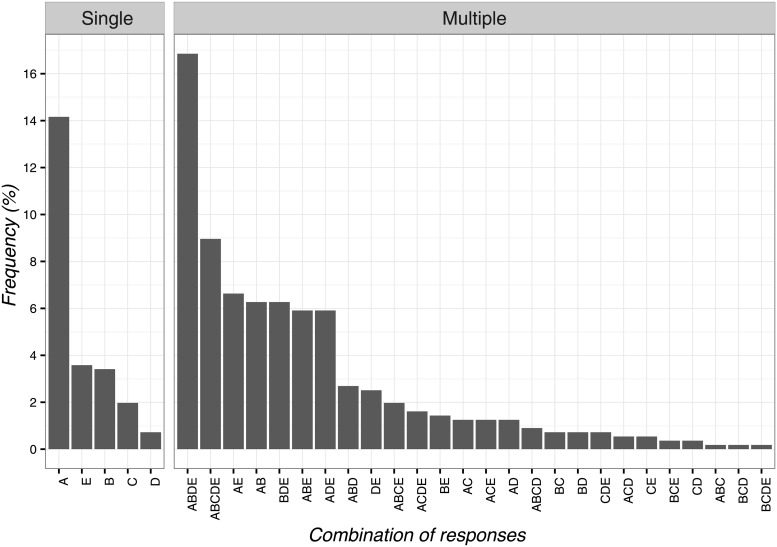
Percentages of responses for not-ethical use of contextual factors. A: it is based on deception, B: it undermines trust between patient and physical therapist, C: the evidence is insufficient, D: legal problems arise, E: it can create adverse effects.

The adoption of CFs was instead considered non-ethical when ‘based on deception’ (n = 79; 14.1%; 95%CI 11.4–17.4) and its combinations with other items as reported in Fig 4. Differently, the least frequent selected answer was when ‘legal problems arise’ (n = 4; 0.7%; 95%CI 0.2–1.9) and its combinations with other items (Fig 4).

### Communication

**Fig 5:**
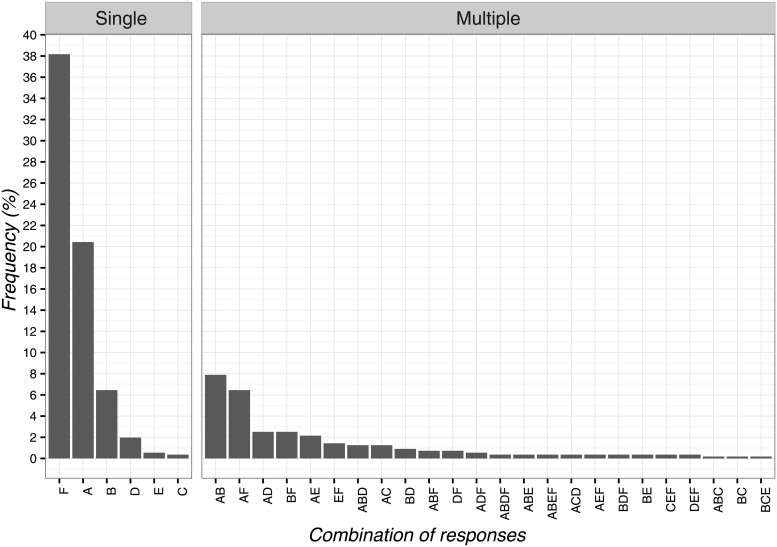
Percentages of responses for communication to patients’ implications of contextual factors. A: it is a treatment that can help and will not hurt, B: it is an effective treatment, C: it is a treatment without a specific effect, D: it is a treatment that induces a psychological change, E: it can help but you are not sure about its effect, F: do not say anything.

When asked about communication and CFs, participants reported a higher frequency of ‘do not say anything’ (n = 213; 38.2%; 95%CI 34.1–42.4). The least frequent chosen item was: ‘it is a treatment without a specific effect’ (n = 2; 0.4%; 95%CI 0.1–1.4). Overall, the combinations of responses are reported in Fig 5.

### Circumstances of CFs application and mechanism of action

As for the circumstances of CFs application, the most frequent item was ‘as an adjunct to other physical therapy interventions to optimize the clinical responses’ (n = 111; 19.9%; 95%CI 16.7–23.5) and its combinations with the response ‘to calm the patient’.

**Fig 6:**
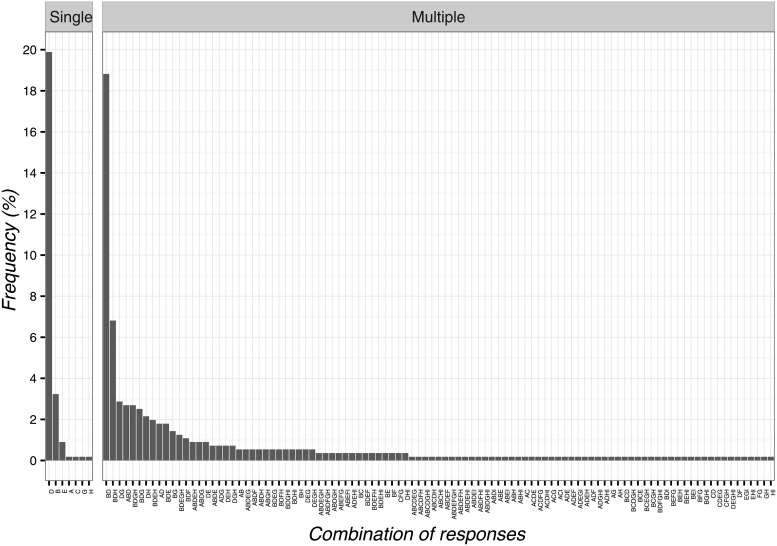
Percentages of responses for circumstances of contextual factors application. A: as a result of unjustified and constant demands for physiotherapy interventions, B: to calm the patient, C: when all other therapies are over, D: as an adjunct to other physical therapy interventions to optimize the clinical responses, E: for non-specific problems, F: to stop patient’s complaints, G: as a diagnostic tool to differentiate between psychological and physiological problems, H: to control pain, I: to gain time.

The least frequent answers were four items: ‘as a result of unjustified and constant demands for physiotherapy interventions’ (n = 1; 0.2%; 95%CI 0.0–1.2), ‘when all other therapies are over’ (n = 1; 0.2%; 95%CI 0.0–1.2), ‘as a diagnostic tool to differentiate between psychological and physiological problems’ (n = 1; 0.2%; 95%CI 0.0–1.2), ‘to control pain’ (n = 1; 0.2%; 95%CI 0.0–1.2). Globally, the combinations of responses are presented in Fig 6.

**Fig 7:**
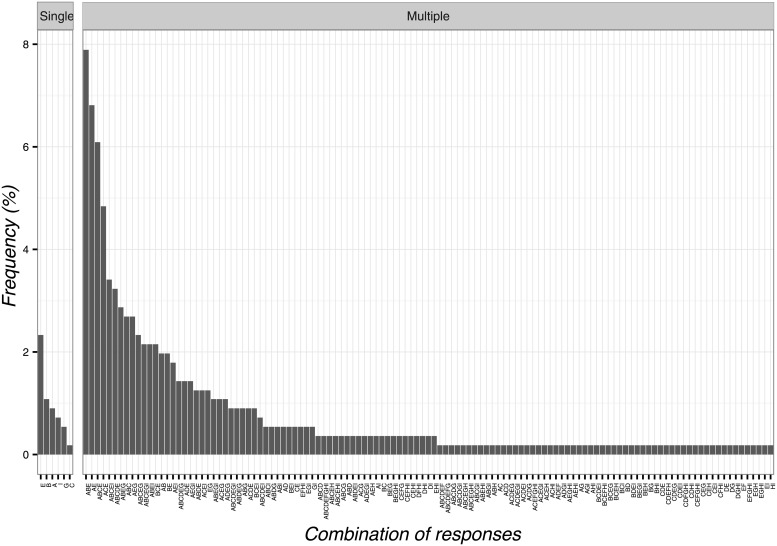
Percentages of responses for contextual factors mechanism of action. A: patient’s expectation, B: conditioning, C: suggestibility, D: natural history of disease, E: psychological factors, F: unexplained, G: physiological/biological factors, H: spiritual energies, I: mind-body connections.

In terms of mechanism of action behind CFs, OMTs selected heterogeneous responses. The most frequent option was ‘psychological factor’ (n = 13; 2.3%; 95%CI 1.3–4.1) and its combination with the items ‘conditioning’ and ‘patient’s expectation’ (Fig 7). The least frequent answer was ‘suggestibility’ (n = 1; 0.2%; 95%CI 0.0–1.2). However, most of the observed frequencies reported by OMTs represented combinations of different answers as presented in Fig 7.

### Correlation between variables

The correlation between the overall frequency and the overall beliefs about CFs was weak (rho = 0.45; p<0.001). Moreover, positive weak associations with Spearman’s rho ≥ 0.40 were found for the following items: uniform (rho = 0.48; p<0.001), patient’s expectation and preference (rho = 0.44; p<0.001), positive attitudes and optimistic behavior (rho = 0.43; p<0.001), and non-verbal communication (rho = 0.40; p<0.001).

**Fig 8:**
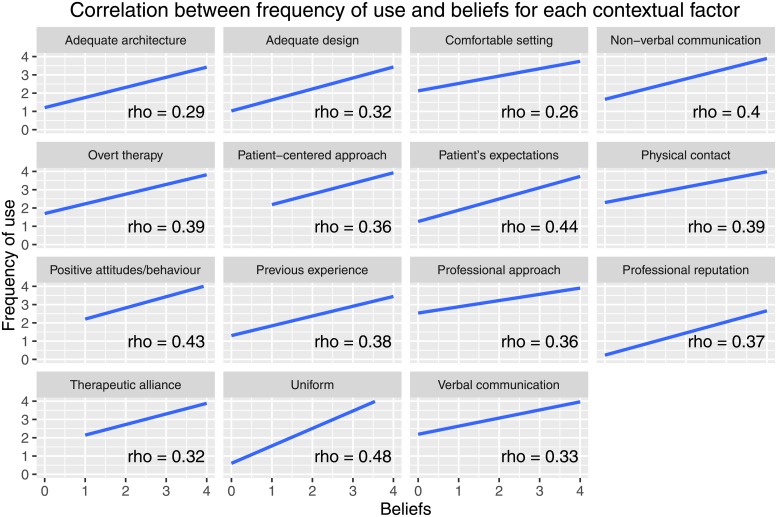
Correlation between frequency of use and beliefs for each contextual factors.

For the other items, the correlation between the frequency of use and the beliefs of each CF was poor with Spearman’s rho < 0.40 as presented in Fig 8.

No significant correlations (Cramer’s V < 0.60) were found between demographic characteristics (section A of the survey) and the responses given (sections B and C of the survey).

## Discussion

To the best of our knowledge, this is the first study that evaluates awareness on CFs among physical therapists specialized in OMTs. The main finding of our research identifies CFs as an aspecific therapeutic intervention capable of influencing patients’ clinical outcome. Moreover, our results suggest that OMTs used and believed in the effectiveness of CFs mainly because of the psychological and physiological therapeutic effects for different healthcare conditions.

As emerged from clinical vignette 1, almost 60% of participants applied TENS. This data inform us about the positive attitude of Italian OMTs towards patients’ expectations in the choice of treatment immediately or after a few days. Indeed, patients’ expectations has been recognized an emerging area of interest in orthopedic physical therapy[56], representing a CF capable of increasing the likelihood of clinical success in low back pain[10]. On the contrary, 40% of participants refused TENS or offered an alternative treatment, thus mirroring the physiotherapists’ behavior reported in previous worldwide surveys[57,58]. Globally, these findings suggest that Italian OMTs consider patients’ expectations as an important therapeutic tool to be integrated in the decision-making process.

Various responses in terms of frequency emerged from clinical vignette 2. The majority of OMTs identified the positive attention of the healthcare team as an explanation for the reduction of pain. This data confirms the importance of the attitude of physical therapists and its influence on musculoskeletal pain, so it should be applied in the clinical context to maximize clinical outcomes[11,15]. Instead, other participants reported an improvement in symptoms connected to the non-organic origin of symptoms, patient’s suggestibility, the natural course of pathology and the patient’s desire to please the physical therapist for their caring. Emerging evidence suggests that these psychobiological components are relevant and have an impact on patients’ pain experience[56,59,60], therefore there is the need to consider and measure these outcomes in patients to potentially enhance the therapeutic effects of CFs in clinical practice.

Although previous studies have not been conducted in the field of orthopaedic physical therapy, some considerations can be made when comparing our results with a similar survey performed among other healthcare professions[19–39]. Similarly to previous surveys among physicians[25,28,29,32], almost 70.0% of OMTs defined CFs as an intervention without a specific effect, but with a possible non-specific effect. These findings reveal that physical therapists conceptualize the context around the treatment as an incidental element that can occur during treatment[61] instead of a powerful therapeutic tool capable of influencing patients’ outcome[56]. This vision could be related to the limited knowledge in academic education on the neurophysiological mechanism underpinning the therapeutic effects of CFs capable of influencing clinical outcomes[6].

At all levels, Italian OMTs reported a high frequency of application of CFs in their daily practice and a strong belief in the actual therapeutic value of CFs, in line with previous studies among physicians[19–34] and nurses[22,37–39].

In detail, the most used and believed CFs were the ones most closely related to patient-physical therapy interaction, representing soft-skills capable of strengthening the patients’ engagement in the therapeutic relationship[62] and to predict the outcome in musculoskeletal physical therapy[11]. These CFs embody a milestone of caring also among nurses. Indeed, in hospital wards nurses spend a significant amount of time with patients, touching them and providing a positive message (e.g., “this pill will decrease your pain”)[63], thus creating the optimal condition for a good therapeutic relationship and for clinical improvement. Instead, physicians often overwork and have less time for interacting and engaging with patients[64], thus reducing the positive therapeutic effects of CFs.

Overall, professional reputation resulted in the least adopted and believed CF and this may depend on a specific lack of knowledge on its clinical relevance. In fact, professional reputation is identified as an undiscovered variable behind the complex concept of professional identity in physical therapy [65], even though it is currently not included in the national academic curriculum as compared to other elements (e.g., verbal and non-verbal communication)[66]. Therefore, there is a need to increase awareness about this CF among physiotherapists, representing an emerging element of personal branding and marketing position[67].

Some specific CFs (e.g., uniform, adequate design and environmental architecture) were overall least believed in, but they were adopted by OMTs with a bimodal frequency (‘daily’ vs ‘never’). The uniform (e.g., white coat) was adopted more in the private sector and in hospitals compared to residential care, representing a CF often imposed in specific settings and the choice of a physical therapist to influence patient’s perception[68]. Moreover, OMTs working in private practice focused more frequently on design and architecture, thus investing economic resources to improve the environment and influence therapeutic outcomes[69]. Instead, in hospitals and in residential care, the low adoption of these CFs may be related to the specificity of these clinical settings, where these elements (e.g., colors of the room, windows and skylights) symbolize infrastructural elements which are not modifiable as compared to music, fragrances or temperature. Therefore, there is a need to direct health policies towards renovating architecture and environmental design with the aim of improving the overall healthcare process and patients’ satisfaction[70].

As for the therapeutic effect of CFs, OMTs believed in ‘psychological and physiological’ effects for most health problems (e.g., pain conditions), thus mirroring a trend previously reported by nurses and physicians who believed in predominantly subjective or a mixture of subjective and objective effects[21,25,27,29,32–35,37]. Less frequently and for other specific clinical conditions, OMTs reported a variety of therapeutic effects as ‘psychological’ (e.g., in oncological conditions) and ‘no benefit’ (e.g., in infectious conditions), depending on the specific health problems considered[18]. Overall, this finding suggests that different effects could explain the therapeutic value of CFs, offering the opportunity to assess them in future research on placebo, nocebo and contextual effect.

OMTs considered the use of ethical implications of CFs as acceptable to enhance positive psychological effects when the clinical experience shows their effectiveness. However, when CFs are based upon deception, they should be avoided to preserve trust between patient and physical therapist, thus highlighting the importance of an ethical application of CFs in the therapeutic session[71]. As reported in previous surveys[18], nurses[22,37] and physicians[19–22,24–29,31–35] were also in favor of the use of placebos and they rarely considered placebos as not allowed or as a treatment that is never permissible.

Furthermore, as previously documented[19,20,22,24–26,29,32–35,37,39], our participants were not used to communicate the adoption of CFs to their patients, nor to inform them that context is an effective addition to the treatment, capable of helping without hurting. The need to disclose to the patient the use of a placebo intervention during the informed consent process is still being debated among clinicians and researchers[72]; however, open-label adoption of a placebo is capable of positively influencing therapeutic outcomes in chronic low back pain[73] and it is appreciated by patients[74].

As clinical indications, OMTs mostly attributed to CFs a therapeutic role in calming patients and as an added strategy to physical therapy interventions, meeting the vision of nurses[22,37] and physicians[20,22,25,27,33] on this topic. Instead, differently from our participants, other healthcare providers offered variable indications[18], thus embracing placebos predominantly to gain a therapeutic advantage, to satisfy the patient’s request, to avoid conflicts, to distinguish organic from psychogenic problems, to control pain, to treat non-specific symptoms, or use when all other interventions have been ineffective[19–22,24,25,27,31–37]. This finding remarks the value of CFs and the need to integrate them in orthopedic physical therapy to enhance therapeutic outcomes[6]. In clinical practice a constant adoption of CFs (e.g., relaxing music, soft light and reassuring voice) along with the best evidence-based treatment[6], offers OMTs the opportunity to manage patients’ symptoms (e.g., fear, avoidance, anxiety) commonly associated with musculoskeletal pain[56].

Moreover, OMTs presented a multifaceted point of view on the mechanisms of action, reporting as most frequent the combination of patient’s expectations, conditioning and psychological factors. This heterogeneity can reflect a lack of knowledge toward the topic as already reported by other healthcare providers[22,24,25,27,28,31,32,34,35], thus suggesting the need of educational efforts on CFs and on placebo and nocebo effects.

The lack of correlations between frequency of use and beliefs can be interpreted under different perspectives: first, OMTs can have some constraints in their clinical practice (e.g., time pressure with a high number of cases to be treated in reduced time) which limit the frequency of CF use despite their beliefs; moreover, their practice may use an evidence-based approach for specific interventions despite general beliefs, thus undermining the implementation of CFs. In this case, improving their preparation by supporting their general beliefs with evidence-based knowledge could be useful.

### Strengths and weaknesses of the study

A high response rate was achieved (61.6%) as compared to previous studies on placebos (from 40.0% to 60.0%)[19–21,25,26], confirming the willingness of Italian OMTs to participate in the survey[44]. A specific group of Italian physical therapists with OMT specializations (n = 906) who are educated to manage mainly musculoskeletal disorders in the private healthcare sector[75] was involved. Therefore, their responses may differ from those of non-specialized physical therapists or from those of other physical therapy specialists[33,75]. Moreover, the are of Italy where respondents are based (North vs Centre and South), less than 10 years of clinical practice, and the majority working full-time may have influenced the participants’ adoption and beliefs of CFs as a therapeutic tool[33,75].

A survey tool was adopted to understand the perspectives of the target population[76]. The questionnaire included different items (e.g., close-ended questions) to increase the likelihood of capturing the complexity of the phenomena under study[77]. Clinical vignettes were used despite their validity being recently questioned in favor of standardized patients as a measure to assess the clinical behavior of physiotherapists[78–80]. However, our methodological choice was based on the impossibility to have a standardized patient for a national online survey, thus mimicking a *modus operandi* previously reported in placebo survey performed on healthcare providers[20].

Given that data were self-reported and retrospective in nature, recall bias can threaten the validity of the findings[20]. Despite the assurance of anonymity, some participants may have misreported their use of CFs[25].

## Conclusions

### Implications for clinicians, policymakers and researchers

A wide use of CFs in physical therapy practice has emerged among Italian OMTs. To ensure appropriate competence, awareness, and the ethical use of the context, this issue should be included in physical therapy graduate and postgraduate study programs and in professional lifelong learning courses. The research on CFs in physical therapy has to be considered in its early stages. Therefore, further quantitative studies evaluating knowledge, uses, and aptitudes on CFs among non-specialized physical therapists across different countries, are strongly recommended. Moreover, studies comparing CFs beliefs, perspectives, and use among healthcare workers are also suggested. To develop a more comprehensive understanding of the phenomena, there is also a need to investigate patients’ perceptions of CFs in physical therapy practice as well as clinicians’ subjective experience of placebo and nocebo effects.

## Supporting information

S1 File The questionnaire: “Knowledge about contextual factors among Italian physical therapists specialized in manual therapy (OMTs)”. (DOCX)

S2 File Il questionario: “Conoscenza dei fattori di contesto tra i fisioterapisti italiani specializzati in terapia manuale ortopedica (OMTs)”. (DOCX)

S3 File Questionnaire dataset. (CSV)
