# Supplementary material for: Physical therapists’ perspectives on using contextual factors in clinical practice: Findings from an Italian national survey
Source: PLoS One. 2018 Nov 30;13(11):e0208159. doi: 10.1371/journal.pone.0208159 (PMC6267986; doi:10.1371/journal.pone.0208159)
Supplement: S1 File — (DOCX) [file pone.0208159.s001.docx]

**Welcome to this survey!**

Dear colleague,

Thank you for taking part in this survey. This survey aims to clarify the use of *contextual factors* in enhancing the therapeutic outcome in physiotherapy.

The contextual factors consist of a series of relational or environmental situations that may affect the perception of patients’ suffering and functional impairment. Examples of the main contextual factors are: words and posture used by the physical therapist, smells, sounds and decor of the therapeutic setting to enhance the effectiveness of physiotherapy.

We consider important to study them in their different applications within the physiotherapy practice.

Please answer the following questions based on your personal clinical experience. Completion of the entire questionnaire is voluntary and will take you 10 to 15 minutes. Your answers are completely anonymous and will only be used for the purposes of this research.

By clicking on the link to the survey, you provide your consent to participate in the study. Whenever you complete the page, click on "Next" to save your answer. If you decide to abandon the survey, click on "Exit".

**Socio-demographic characteristics**

***What is your gender?*** *[select]*

- Male
- Female

***How old are you?*** *[complete]*

……….

***How long have you been licensed as a physical therapist?*** *[complete]*

……….

***Which part of Italy do you work in?*** *[select]*

- North
- Center
- South

***What is your clinical workplace?*** *[select]*

- Hospital
- Residential care (nursing home, RSA)
- Private practice

***What is your field of intervention?*** *[select]*

- Geriatric
- Neurological
- Musculoskeletal
- Hearth, Respiratory, Pediatric

***How many hours do you work each week?*** *[select]*

- 0-15
- 16-30
- 31-45
- 46-60
- > 60

**Clinical vignettes**

**Clinical vignette 1**

A 40-year-old man, freelancer, visits your clinic complaining about low back pain. He requests TENS therapy to return to work more quickly. Based on clinical examination you don’t find contraindications for the use of TENS, but you know that in this case there is no indication to use this therapy. The patient insists on requiring TENS on the grounds that this therapy helped him in the past during a previous episode of low back pain.

***What would you do in this situation?*** *[select]*

- (A) deliver TENS
- (B) tell the patient that low back pain will resolve itself in a few days
- (C) suggest the possibility of delivering TENS if the clinical condition fails to improve
- (D) advise a follow-up appointment in the following days
- (E) advise a different treatment commonly used for low back pain
- (F) try to convince the patient of the usefulness of TENS

**Clinical vignette 2**

In a hospital, a patient with important shoulder pain receives TENS therapy on demand, several times a day. In case of frequent requests therapy is occasionally replaced with sham T.E.N.S. (with power-off). The patient reports that in each case TENS (whether active or sham) improve their clinical condition.

***What conclusion can be drawn on the effectiveness of TENS sham?*** *[More answers are possible]*

- (A) the positive attention of the healthcare team leads to decreased pain
- (B) pain is not organic but psychological
- (C) the patient is very suggestible
- (D) pain intensity decreases naturally
- (E) the patient provides the response expected by the physical therapist

**Frequency of use (1/2)**

***How often in your career have you intentionally used contextual factors to enhance the result of physiotherapy?*** *[select]*

- many times
- often
- at least once
- never

**Frequency of use (2/2)**

***State how often you have intentionally used the following contextual factors with a patient to improve the outcome of physiotherapy:*** *[select]*

|  | **Every day** | **Around once a week** | **Around once a month** | **Around once a year** | **Never** | **I was not aware it was a contextual factor capable of influencing the therapeutic outcome** |
| --- | --- | --- | --- | --- | --- | --- |
| Professional reputation |  |  |  |  |  |  |
| Uniform |  |  |  |  |  |  |
| Positive attitudes and optimistic behavior |  |  |  |  |  |  |
| Patient’s expectation and preference |  |  |  |  |  |  |
| Patient’s previous experience |  |  |  |  |  |  |
| Verbal communication |  |  |  |  |  |  |
| Non-verbal communication |  |  |  |  |  |  |
| Empathetic therapeutic alliance with the patient |  |  |  |  |  |  |
| Overt therapy |  |  |  |  |  |  |
| Patient-centered approach |  |  |  |  |  |  |
| Professional approach to patient |  |  |  |  |  |  |
| Physical contact with the patient |  |  |  |  |  |  |
| Comfortable setting |  |  |  |  |  |  |
| Adequate environmental architecture |  |  |  |  |  |  |
| Adequate design |  |  |  |  |  |  |

**Beliefs**

***How much do you believe that your therapeutic outcome can be influenced by ...?*** *[select]*

|  | **Very Much** | **Much** | **Enough** | **Few** | **None** | **I don’t know** |
| --- | --- | --- | --- | --- | --- | --- |
| Professional reputation |  |  |  |  |  |  |
| Uniform |  |  |  |  |  |  |
| Positive attitudes and optimistic behavior |  |  |  |  |  |  |
| Patient’s expectation and preference |  |  |  |  |  |  |
| Patient’s previous experience |  |  |  |  |  |  |
| Verbal communication |  |  |  |  |  |  |
| Non-verbal communication |  |  |  |  |  |  |
| Empathetic therapeutic alliance with the patient |  |  |  |  |  |  |
| Overt therapy |  |  |  |  |  |  |
| Patient-centered approach |  |  |  |  |  |  |
| Professional approach to patient |  |  |  |  |  |  |
| Physical contact with the patient |  |  |  |  |  |  |
| Comfortable setting |  |  |  |  |  |  |
| Adequate environmental architecture |  |  |  |  |  |  |
| Adequate design |  |  |  |  |  |  |

**Ethical issues**

***The use of contextual factors for therapeutic purposes can be considered ethically acceptable when ...*** *[you can select more than one answer]*

- (A) it exerts beneficial psychological effects
- (B) the other therapies are over
- (C) the patient wants or expects this treatment
- (D) clinical experience has shown their effectiveness

***The use of contextual factors for therapeutic purposes can be considered ethically unacceptable when ...*** *[you can select more than one answer]*

- (A) it is based on deception
- (B) it undermines trust between patient and physical therapist
- (C) evidence is insufficient
- (D) legal problems arise
- (E) it can create adverse effects

**Communication and application**

***How do you communicate to the patient the use of contextual factors at the end of treatment? You tell them that ...*** *[you can select multiple possibilities]*

- (A) it is a treatment that can help and will not hurt
- (B) it is an effective treatment
- (C) you do not say anything
- (D) it is a treatment without a specific effect
- (E) it is a treatment that induces a psychological change
- (F) it can help but you are not sure about its effect.

***Under what circumstances would you use contextual factors?*** *[you can select multiple possibilities]*

- (A) as a result of unjustified and constant demands for physiotherapy interventions
- (B) to calm the patient
- (C) when all other therapies are over
- (D) as an adjunct to other physical therapy interventions to optimize clinical responses
- (E) for non-specific problems
- (F) to stop the patient’s complaints
- (G) as a diagnostic tool to differentiate between psychological and physiological problems
- (H) to control pain
- (I) to gain time

**Mechanism of action, therapeutic effect and definition**

***What mechanisms of action can explain the effect of contextual factors?*** *[you can select multiple possibilities]*

- (A) patient’s expectation
- (B) conditioning
- (C) suggestibility
- (D) natural history of disease
- (E) psychological factors
- (F) unexplained
- (G) physiological/biological factors
- (H) spiritual energies
- (I) mind-body connections

***What are, in your opinion, the potential effects of contextual factors in the following health problems?*** *[select]*

|  | **Psychological** | **Physiological** | **Psychological and Physiological** | **No benefit** |
| --- | --- | --- | --- | --- |
| acute pain |  |  |  |  |
| chronic pain |  |  |  |  |
| cognitive disorder |  |  |  |  |
| emotional disorder |  |  |  |  |
| gastrointestinal disorder |  |  |  |  |
| sexual disorder |  |  |  |  |
| drug and medication addiction |  |  |  |  |
| neurological disorder |  |  |  |  |
| rheumatologic disorder |  |  |  |  |
| immune/allergic disorder |  |  |  |  |
| oncological disorder |  |  |  |  |
| cardiovascular disorder |  |  |  |  |
| infections |  |  |  |  |
| insomnia |  |  |  |  |

***How would you define, in the light of this survey, the therapeutic role of contextual factors?*** *[select]*

- an intervention without a specific effect for the condition being treated, but with a possible aspecific effect
- an intervention that has a special effect through known physiological mechanisms
- sham treatment used as control tests for safety and efficacy of active treatment
- a harmless or inert intervention

***Dear colleague, thanks for taking the time to complete this survey!***
