# Supplementary material for: Physical therapists’ perspectives on using contextual factors in clinical practice: Findings from an Italian national survey
Source: PLoS One. 2018 Nov 30;13(11):e0208159. doi: 10.1371/journal.pone.0208159 (PMC6267986; doi:10.1371/journal.pone.0208159)
Supplement: S2 File — (DOCX) [file pone.0208159.s002.docx]

**Benvenuto a questo questionario!**

Gentile collega grazie per prendere parte a questo questionario.

Questa indagine serve a chiarire l’utilizzo dei *fattori di contesto* per il potenziamento del risultato terapeutico nell’attività clinica fisioterapica.

I fattori di contesto sono costituiti da una serie di situazioni relazionali o ambientali che possono influenzare la percezione che il paziente ha della sua condizione di sofferenza e di limitazione funzionale. Esempi dei principali fattori di contesto sono: le parole e la postura utilizzata dal terapista, gli odori, i suoni e l’arredamento del setting terapeutico. Noi consideriamo importante studiarli nella pratica clinica quotidiana del fisioterapista.

Gentilmente rispondi alle seguenti domande sulla base della tua personale esperienza e pratica clinica. La compilazione dell’intero questionario è volontaria e richiede 10-15 minuti. Le tue risposte sono completamente anonime e saranno utilizzate solamente per gli scopi di questa ricerca.

Cliccando sul link del questionario, tu fornisci il tuo consenso a partecipare allo studio. Quando completi la pagina, clicca su “Prosegui” per salvare le tue risposte. Se decidi di abbandonare il questionario, seleziona “Uscita”.

**Caratteristiche socio-demografiche**

***Quale è il tuo sesso?*** *[seleziona]*

- Maschio
- Femmina

***Quanti anni hai?*** *[completa]*

……….

***Da quanti anni lavori come fisioterapista?*** *[completa]*

……….

***In che regione dell’Italia lavori?*** *[seleziona]*

- Nord
- Centro
- Sud

***Quale è il tuo ambito lavorativo?*** *[seleziona]*

- Ospedale
- Strutture assistenziali (casa di riposo, RSA)
- Ambulatorio – struttura privata

***Quale è il tuo campo di intervento?*** *[seleziona]*

- Geriatrico
- Neurologico
- Muscoloscheletrico
- Cardiaco, respiratorio, pediatrico

***Quante ore lavori per ogni settimana?*** *[seleziona]*

- 0-15
- 16-30
- 31-45
- 46-60
- > 60

**Scenario clinico**

**Scenario clinico 1**

Un ragazzo di 40 anni libero professionista viene in visita nel tuo ambulatorio lamentando un dolore lombare. Lui chiede una terapia con la TENS per poter tornare a lavorare più rapidamente. Sulla base dell’esame clinico, non riscontri controindicazioni all’utilizzo della TENS, ma sai che in questo caso non ci sono indicazioni per utilizzare questa terapia. Il paziente insiste convintamente a richiedere di essere trattato con TENS, basandosi sul fatto che questa lo ha aiutato in passato durante un precedente episodio di lombalgia.

***Che cosa faresti in questa situazione?*** *[seleziona]*

- (A) eroghi la TENS
- (B) dici al paziente che la lombalgia si risolverà in qualche giorno
- (C) proponi la possibilità di erogare la TENS successivamente qualora la condizione clinica non migliori
- (D) proponi un appuntamento di follow up nei giorni successivi
- (E) proponi un trattamento diverso comunemente utilizzato per la lombalgia
- (F) provi a convincere il paziente che non necessita di TENS

**Scenario clinico 2**

In un ospedale, un paziente con dolore importante di spalla riceve TENS terapia più volte al giorno su richiesta. Per le frequenti richieste, occasionalmente la terapia viene sostituita con TENS sham (con apparecchio spento). Il paziente riporta che in ogni caso la TENS (sia attiva che sham) ha dato buoni risultati.

***Che conclusione puoi trarre circa l’efficacia della TENS sham?*** *[Più risposte sono possibili]*

- (A) l’attenzione positiva dello staff sanitario conduce alla diminuzione del dolore
- (B) il dolore non ha cause organiche, ma psicologiche
- (C) il paziente è molto suggestionabile
- (D) l’intensità del dolore diminuisce naturalmente
- (E) il paziente fornisce la risposta che tu come operatore vorresti sentirti dire

**Frequenza di utilizzo (1/2)**

***Con che frequenza nella tua carriera hai volutamente utilizzato i fattori di contesto per potenziare il risultato terapeutico?*** *[seleziona]*

- molte volte
- spesso
- almeno una volta
- mai

**Frequenza di utilizzo (2/2)**

***Indica con che frequenza hai volutamente utilizzato i seguenti fattori di contesto con il paziente per potenziare il risultato terapeutico:*** *[seleziona]*

|  | **Ogni giorno** | **Almeno una volta a settimana** | **Almeno una volta al mese** | **Almeno una volta all’anno** | **Mai** | **Non pensavo fosse un fattore di contesto capace di influenzare l’esito della terapia** |
| --- | --- | --- | --- | --- | --- | --- |
| La tua reputazione professionale |  |  |  |  |  |  |
| L’uniforme |  |  |  |  |  |  |
| Gli atteggiamenti e comportamenti positivi con il paziente |  |  |  |  |  |  |
| Le aspettative e le preferenze del paziente |  |  |  |  |  |  |
| Le precedenti esperienze del paziente |  |  |  |  |  |  |
| La comunicazione verbale |  |  |  |  |  |  |
| La comunicazione non verbale |  |  |  |  |  |  |
| L’alleanza terapeutica empatica con il paziente |  |  |  |  |  |  |
| La terapia resa evidente |  |  |  |  |  |  |
| L’approccio centrato sul paziente |  |  |  |  |  |  |
| L’approccio professionale con il paziente |  |  |  |  |  |  |
| Il contatto fisico con il paziente |  |  |  |  |  |  |
| L’ambiente confortevole |  |  |  |  |  |  |
| L’architettura adeguata |  |  |  |  |  |  |
| Il design ambientale accurato |  |  |  |  |  |  |

**Credenze**

***Quanto credi che il tuo risultato terapeutico possa essere influenzato da…?*** *[seleziona]*

|  | **Moltissimo** | **Molto** | **Abbastanza** | **Poco** | **Per nulla** | **Non so** |
| --- | --- | --- | --- | --- | --- | --- |
| La tua reputazione professionale |  |  |  |  |  |  |
| L’uniforme |  |  |  |  |  |  |
| Gli atteggiamenti e comportamenti positivi con il paziente |  |  |  |  |  |  |
| Le aspettative e le preferenze del paziente |  |  |  |  |  |  |
| Le precedenti esperienze del paziente |  |  |  |  |  |  |
| La comunicazione verbale |  |  |  |  |  |  |
| La comunicazione non verbale |  |  |  |  |  |  |
| L’alleanza terapeutica empatica con il paziente |  |  |  |  |  |  |
| La terapia resa evidente |  |  |  |  |  |  |
| L’approccio centrato sul paziente |  |  |  |  |  |  |
| L’approccio professionale con il paziente |  |  |  |  |  |  |
| Il contatto fisico con il paziente |  |  |  |  |  |  |
| L’ambiente confortevole |  |  |  |  |  |  |
| L’architettura adeguata |  |  |  |  |  |  |
| Il design ambientale accurato |  |  |  |  |  |  |

**Aspetti etici**

***L’utilizzo dei fattori di contesto a fini terapeutici può essere considerato eticamente accettabile quando...*** *[puoi selezionare più di una risposta]*

- (A) induce effetti psicologici benefici
- (B) tutte le altre terapie sono terminate
- (C) il paziente vuole o aspetta quel trattamento
- (D) l’esperienza clinica ne ha dimostrato l’efficacia

***L’utilizzo dei fattori di contesto a fini terapeutici può essere considerato eticamente non accettabile quando...*** *[puoi selezionare più di una risposta]*

- (A) è basato sull’inganno
- (B) mina la fiducia tra fisioterapista e paziente
- (C) le prove di efficacia non sono sufficienti
- (D) emergono problemi legali
- (E) può creare effetti avversi

**Comunicazione ed applicazione**

***Come comunichi al paziente l’utilizzo dei fattori di contesto a fine di trattamento? Gli dici che ...*** *[puoi selezionare più di una risposta]*

- (A) è un trattamento che può aiutare e non farà male
- (B) è un trattamento efficace
- (C) non dici nulla
- (D) è un trattamento senza un effetto specifico
- (E) è un trattamento che induce modifiche psicologiche
- (F) è un trattamento che può aiutare, ma non sei sicuro di come agisca.

***In che circostanze applicheresti i fattori di contesto?*** *[puoi selezionare più di una risposta]*

- (A) a seguito di “ingiustificate e continue” richieste di fisioterapia
- (B) per calmare il paziente
- (C) quando tutte le altre terapie sono terminate
- (D) come aggiunta agli altri interventi di fisioterapia per ottimizzare la risposta clinica
- (E) per problemi non specifici
- (F) per portare il paziente a smettere di lamentarsi
- (G) come strumento diagnostico per discriminare un problema di tipo psicologico da uno organico
- (H) per controllare il dolore
- (I) per guadagnare tempo

**Meccanismo di azione, effetto terapeutico e definizione**

***Quali meccanismi d’azione possono spiegare l’effetto dei fattori di contesto?*** *[puoi selezionare più di una risposta]*

- (A) le aspettative del paziente
- (B) il condizionamento
- (C) la suggestionabilità
- (D) la storia naturale del disturbo
- (E) i fattori psicologici
- (F) non spiegabile
- (G) fattori fisiologici/biologici
- (H) energie spirituali
- (I) connessione corpo-mente

***Quali sono, secondo te, i potenziali effetti dei fattori di contesto nei seguenti problemi di salute?*** *[seleziona]*

|  | **Psicologico** | **Fisiologico** | **Fisiologico e psicologico** | **Nessun beneficio** |
| --- | --- | --- | --- | --- |
| Dolore acuto |  |  |  |  |
| Dolore cronico |  |  |  |  |
| Problemi cognitivi |  |  |  |  |
| Problemi emozionali |  |  |  |  |
| Problemi gastrointestinali |  |  |  |  |
| Problemi sessuali |  |  |  |  |
| Dipendenze da droghe o farmaci |  |  |  |  |
| Problemi neurologici |  |  |  |  |
| Problemi reumatologici |  |  |  |  |
| Problemi immunitari/allergie |  |  |  |  |
| Problemi oncologici |  |  |  |  |
| Problemi cardiovascolari |  |  |  |  |
| Infezioni |  |  |  |  |
| Insonnia |  |  |  |  |

***Come definiresti, alla luce di questa indagine il ruolo terapeutico dei fattori di contesto?*** *[seleziona]*

- un intervento senza un effetto specifico per la condizione trattata, ma con un possibile effetto aspecifico
- un intervento che ha un effetto specifico attraverso meccanismi fisiologici noti
- un intervento simulato usato come test di controllo per la sicurezza e l’efficacia di un trattamento attivo
- un intervento che è innocuo o inerte

***Gentile collega grazie per averci dedicato il tempo per compilare il questionario!***
